# Artificial Intelligence in Otology, Rhinology, and Laryngology: A Narrative Review of Its Current and Evolving Picture

**DOI:** 10.7759/cureus.66036

**Published:** 2024-08-02

**Authors:** Ayushi Ghosh Moulic, Sagar S Gaurkar, Prasad T Deshmukh

**Affiliations:** 1 Otolaryngology - Head and Neck Surgery, Jawaharlal Nehru Medical College, Datta Meghe Institute of Higher Education and Research, Wardha, IND

**Keywords:** deep learning, machine learning, rhinology, otology, artificial intelligence

## Abstract

With technological advancements, artificial intelligence (AI) has progressed to become a ubiquitous part of human life. Its aspects in otorhinolaryngology are varied and are continuously evolving. Currently, AI has applications in hearing aids, imaging technologies, interpretation of auditory brain stem systems, and many more in otology. In rhinology, AI is seen to impact navigation, robotic surgeries, and the determination of various anomalies. Detection of voice pathologies and imaging are some areas of laryngology where AI is being used. This review gives an outlook on the diverse elements, applications, and advancements of AI in otorhinolaryngology. The various subfields of AI including machine learning, neural networks, and deep learning are also discussed. Clinical integration of AI and otorhinolaryngology has immense potential to revolutionize the healthcare system and improve the standards of patient care. The current applications of AI and its future scopes in developing this field are highlighted in this review.

## Introduction and background

Artificial Intelligence (AI) encompasses computer algorithms designed to mechanize the process of cognition. Highly functional modeling and computational techniques are used for the analysis of data sets and execution of various tasks, including visual perception, word and object recognition, making decisions, and operation of autonomous vehicles [1]. The subfields of AI that are primal to healthcare include machine learning and natural learning processing [1]. Machine learning further branches out into supervised learning, unsupervised learning, and deep learning [2].

Analysis of human-labeled data to produce a set function by machine learning algorithms and thus predicting the label for unfamiliar data is referred to as supervised learning. This approach is beneficial for predicting known outcomes in various problems. On the other hand, the algorithms that help in learning patterns within the data are referred to as unsupervised learning [1]. Deep learning refers to using deep neural networks with multiple hidden layers, which are highly effective in solving complex machine learning problems like image and speech recognition [3]. Natural language processing involves the analysis of text or speech to enable computer algorithms to access information. It aims to facilitate human-computer interaction using ordinary language [1]. 

This review aims to integrate the various branches of AI used in the field of otorhinolaryngology and compile them together along with their particular implications in otology, rhinology, laryngology, and head and neck and also discuss the evolution, current application, and further advancements.

## Review

Methodology

The methodology involved a narrative literature search strategy using multiple electronic databases, including PubMed, Scopus, and Google Scholar. We used the keywords and Medical Subject Headings (MeSH) terms "artificial intelligence," "otology," "rhinology," "laryngology," "machine learning," "deep learning," and "convolutional neural networks" in combination. We included review and original articles related to otolaryngology based on the presence of these terms in the title or abstract. Case reports and editorials were however excluded.

The different aspects and current applications of AI in otology, rhinology, and laryngology are discussed separately in chronological order, with a separate note on the futuristic applications of AI in otorhinolaryngology.

Artificial intelligence in otology

Impact of AI on Hearing Aid Technologies

The Siemens Centra (made in 2006 by Siemens AG, Munich, Germany) was the first built-in machine-learned hearing aid produced commercially [4]. Adjustments to the volume control and gradual default settings at power-up help Centra learn the individual's preferred gains [5]. Hearing aids can now be integrated with smartphones, utilizing user-friendly communication abilities with enhanced sensing and processing utilities [6]. Hearing aids can detect human words, translate languages in real-time, detect falls, and monitor the patient's overall well-being [7].

Vestibular Rehabilitation Using AI

The application of AI in vestibular manifestations and vertigo ranges from diagnosis and classification to the current application of virtual reality rehabilitation. The expert system developed by Mira et al., ‘Vertigo’, was one of the first technologies to incorporate AI to diagnose causes of dizziness in 200 subjects [8]. It used case histories and neurological examinations to confirm or reject a diagnosis for the cause of dizziness [8]. Otoneurological expert systems, first developed in 1999, were developed to establish a diagnosis and enhance the user's knowledge [9]. A machine learning named Galactica was used by Laurikkala et al. in their study and generated diagnostic decision rules using data from 564 vertigo patients, primarily diagnosed with Menière's disease, vestibular schwannoma, traumatic vertigo, sudden deafness, benign paroxysmal positional vertigo (BPPV), and vestibular neuritis [10].

The EMBalance diagnostic platform surpasses current methods by classifying patients into diagnostic categories and providing a recommendation tool for general practitioners to request the necessary information for diagnosis. Thanks to multiple data mining models, it can offer multiple diagnoses per patient. Additionally, while previous systems used 10-240 features for training and testing, EMBalance uses around 350 features to characterize patients [11].

Vestibular rehabilitation is the most important management of imbalance and order disorders. Apart from older methods, a virtual-reality (VR) rehabilitation program based on interactive gaming and recording based on sensors has also been introduced [12]. Physical therapists can tailor the content of the games and levels of difficulty to match the patients' life scenarios. Optical tracking and skeleton-based detection libraries, once advancements in vision-based technology from Microsoft Kinect (Microsoft Corporation, Redmond, Washington, United States), captured extensive human movement, reducing the need for manual intervention in rehabilitative training. With the discontinuation of Kinect, these technologies have evolved and continue to be applied in other devices. These capture the extensive movement of humans, reducing the need for manual intervention in rehabilitative training. Rehabilitation systems should offer immediate visual, auditory, and tactile feedback to enhance patient engagement and improve training effectiveness [12].

Auditory Brainstem Response and AI

To detect hearing deficits in individuals with learning disability, global developmental delays, and newborns, auditory brain stem response is the basic tool [4]. However, the interpretation of waveforms is mostly through the opinion of clinicians and may be inconsistent [4]. Several studies have discussed models classifying the waveforms of ABR with an average accuracy of 90.74% in differentiating the normal and abnormal signals [13]. However, a study presented a neural network model to classify 190 paired auditory brainstem responses (ABR) into three categories. The responses were recorded as "clear," "inconclusive," or "absent,” with high rates of accuracy (92.9%), sensitivity (92.9%), and specificity [14]. In a study in 2023, the ABR image was standardized using a deep learning network model, and the accuracy was 84.9% [15]. Applying a model like this in the future may provide real-time assistance to clinicians and offer a more objective interpretation of auditory brainstem responses [4].

Imaging Modalities and AI in Otology

Imaging is the most important modality to not only reach a diagnosis but also for academic purposes. Medical imaging can be grouped into classification, segmentation, and combined methods to study middle ear diseases. Convolutional neural networks (CNN) refer to a deep neural network applied to image recognition, classification, semantic segmentation, and instant segmentation [16]. In a study by Myburgh et al., machine learning was employed by using an Android smartphone with an internet connection to expand the automatic diagnostic system [17]. Segmentation refers to extracting areas of interest in the given images [18]. The image data produced by endoscopy is used for segmentation in otolaryngology. To diagnose middle ear illnesses and or study the interior of the tympanic membrane or middle ear in detail, studies focus on the diseased tympanic membrane [19]. In some studies, image segmentation was done by semi-automatedly marking the region of interest [20,21]. A complete automatic segmentation of the tympanic membrane and its diseases was done by Pham et al. [22]. Efforts to improve diagnostic performance often involve using segmented images in classification research. This implicated identifying the interested area while focusing on the distinguishing feature during the disease classification [19]. A novel hybrid system called otitis media (OM) computer-aided detection (CAD) was developed by Shie et al., which used machine learning to segment the tympanic membrane to classify various forms of otitis media [23]. CNN has great potential to unravel the multifaceted implications of AI in further automating the classification and segmentation process of image recognition. It can be used for many other purposes in imaging modalities in otorhinolaryngology.

Artificial intelligence in rhinology

Diagnosis and Differentiation of Benign and Malignant Diseases

Deep learning is used for image segmentation and classification [24]. CNN was used by Liu et al. to distinguish between inverted papilloma and inverted papilloma with malignant transformation with 77.9% accuracy [25]. Li et al. also used CNN to form a detection model for nasopharyngeal carcinoma, which performed the task in a much shorter duration [26]. Sino-nasal malignancies can be detected using deep learning by utilizing the CT and MRI texture in the preoperative setting to check the extent of the disease and develop a proper surgical plan [27,28]. Diagnosing various anatomical variations and automated classification of the stomatal complex were also observed using the Google Inception-V3 CNN [29].

Categorization of Chronic Rhinosinusitis

The tendency for diseases to cluster based on observations is a key characteristic of unsupervised learning. This process identifies patterns by examining data without needing labels for each data point [30]. Unsupervised learning has been used in various studies to identify patient clusters and categorize them into various classes of chronic rhinosinusitis. Parcel et al. used the Gower distance to combine 22 variables in forming a cluster, including demographics, patient-reported quality of life (PRQOL), and comorbidities [31]. Similarly, Divekar et al., in their study, used a Force-directed algorithm (ForceAtlas) for symptom-based clustering [32].

Surgical Assessment, Navigation, and Robotics

The introduction of AI in surgical rhinology has been an incredible milestone. Image guidance surgery has evidence of better surgical outcomes due to improved dissection and lesser injury risk to nearby structures [33]. Reiter et al. introduced a new concept of endoscopic CT following a video-CT algorithm involving Structure from Motion (SfM), a CNN technique to form a photo-realistic three-dimensional (3D) reconstruction of the surgical site [34]. Research on deep-learning-based endoscopic surgery has been extended by Biek et al. by a natural language processing used to navigate the future endoscopic position with workflow annotations [35]. Robotic surgeries have made significant progress in other otolaryngological fields with oropharyngeal tumors, thyroid, and other surgeries. Steinhart et al. introduced robotic surgery in the paranasal sinus and were able to perform the resection of the anterior ethmoid sinus [36]. Newer advances in robotic surgery may include automated features using machine learning algorithms and miniature instruments [24,37].

Artificial intelligence in laryngology

Image-Based Diagnosis

Laryngoscopic images have been used along with deep convoluted neural networks to detect all times of laryngeal lesions as well as for comparison between malignant and pre-malignant lesions. Witt et al. used an artificial neural network (ANN) to compare the texture and hue between the laryngoscopic images of non-laryngopharyngeal reflux and laryngopharyngeal reflux disease [38,39]. A study by Cho et al. compared various CNN models to determine the accuracy of normal vocal cords. The clinical applications of deep learning models in laryngoscopy can be estimated by a real-time classification of a video stream using a combination of the VGG16 model, OpenCV, and Grad-CAM (Gradient-weighted Class Activation Mapping) [40]. AI has also shown extremely high accuracy in assessing laryngeal images, identifying healthy tissue, and differentiating between malignant and benign lesions [38].

Detection of Abnormalities of Voice

In pathological manifestations of speech and voice, AI-based methods for identifying voice disorders involve using algorithms that analyze speech patterns and detect abnormalities. This involves evaluating features like pitch, loudness, and voice quality and comparing them to a database of typical speech patterns [41]. Speech analysis systems for voice pathology have utilized various machine learning algorithms, such as Decision Tree (DT), Extreme Learning Machine (ELM), Naïve Bayes (NB), and Support Vector Machine (SVM). These algorithms have proven the effective and efficient classification and differentiation of pathological and normal voices [41,42].

Future scopes of AI in otorhinolaryngology

A study has introduced computational audiology as a futuristic approach for a diagnostic and rehabilitative approach to lessen the burden of global hearing loss [43]. Synergistic application of machine-based auditory profiling like Audiogene (Center for Bioinformatics and Computational Biology at The University of Iowa, United States) with cochlear modeling can be used to determine the probability of hearing loss in potential candidates. It can also help establish treatment and rehabilitation protocols [43-45]. Using deep learning neural networks, ultra-high resolution CT images of the cochlea can help in segmentation and further assist in planning cochlear surgeries [46]. The application of 3D virtual reality in rehabilitating vestibular disorders and vertigo is the futuristic application of AI in otology [47]. In rhinology, advancements are expected in surgical fields, especially rhinoplasty. Machine learning with 3D morphism deep learning can assess the geometrical features and be used to detect the different surgical approaches and post-surgical outcomes [48]. Real-time face mapping and navigation during the surgery can prevent intraoperative and postoperative complications. Many developments are still being made regarding detecting and managing vocal pathologies, including developing a mobile system to detect voice pathology using machine-learning algorithms [49]. Therefore, there is high hope for future AI endeavors in otorhinolaryngology. A summary of the included articles is given in Table 1.

**Table 1:** Summary of included articles 3D: three-dimensional; VR: virtual reality; CNN: convolutional neural networks; AI: artificial intelligence; ML: machine learning; ANN: artificial neural network

Author Name	Year of Study	Study Objectives
Mira et al. [8]	1990	Exploration of expert systems as a diagnostic aid in otoneurology.
Kentala et al. [9]	1999	Development of an otoneurological expert system for diagnosing vertigo.
Laurikkala et al. [10]	2001	Application of a novel machine learning program to discover otological diagnoses.
Hsu et al. [20]	2004	Development of a computer program to calculate the size of tympanic membrane perforations.
Steinhart et al. [36]	2004	Application of a new robotic system for paranasal sinus surgery.
Mueller et al. [5]	2008	Investigates the use of trainable hearing aids to examine real-world preferred gain settings.
Hildebrand et al. [45]	2009	AudioGene Audioprofiling, a machine-based candidate gene prediction tool for non-syndromic hearing loss.
Ibekwe et al. [21]	2009	Quantitative analysis method for tympanic membrane perforation that is simple and reliable.
Yeh et al. [12]	2014	Development of a machine learning-based tool for assessing imbalance and vestibular dysfunction with a VR rehabilitation system.
Shie et al. [23]	2014	Hybrid feature-based segmentation and classification system for computer-aided self-diagnosis of otitis media.
Norouzi et al. [18]	2014	Review of medical image segmentation methods, algorithms, and applications.
Witt et al. [39]	2014	Detection of chronic laryngitis using color and texture analysis of laryngoscopic images.
LeCun et al. [30]	2015	Overview of deep learning, its methodologies, and applications.
Divekar et al. [32]	2015	Symptom-based clustering in chronic rhinosinusitis and its relation to aspirin sensitivity and postsurgical outcomes.
Reiter et al. [34]	2016	Learning-based photometric reconstruction for endoscopic sinus surgery.
Sandhya et al. [13]	2016	Classification of Brainstem Auditory Evoked Potentials using ANN based on time and frequency domain features.
Aldaz et al. [6]	2016	Development of a smartphone-based system for learning and inferring hearing aid settings.
Exarchos et al. [11]	2016	Mining balance disorders’ data for developing diagnostic decision support systems.
van Gerven et al. [3]	2017	Discusses the use of artificial neural networks as models for neural information processing.
Ramkumar et al. [28]	2017	MRI-based texture analysis to differentiate sinonasal squamous cell carcinoma from inverted papilloma.
Myburgh et al. [17]	2018	Development of a low-cost automated smartphone- and cloud-based otitis media diagnosis system.
Li et al. [26]	2018	Development of a deep learning model for detecting nasopharyngeal malignancies using endoscopic images.
Verhulst et al., [44]	2018	Computational modeling of the human auditory periphery, including responses and hearing loss.
Verde et al., [49]	2019	Use of a mobile app leveraging AI to improve voice disorder identification.
Wolfgang et al. [7]	2019	AI and ML are advancing hearing technology, improving diagnostics and treatment outcomes.
Bur et al. [1]	2019	Review of AI applications in Otolaryngology highlighting its current state and potential benefits.
McKearney et al. [14]	2019	Objective auditory brainstem response classification using machine learning.
Chowdhury et al. [29]	2019	Automated classification of osteomeatal complex inflammation on CT using convolutional neural networks.
Żurek et al. [38]	2022	Systematic review and meta-analysis of AI in laryngeal endoscopy.
Cho et al. [40]	2022	Comparison of CNN models for determining vocal fold normality in laryngoscopic images.
Ur et al. [41]	2024	Voice disorder detection using machine learning algorithms for speech and language pathology.
Al-Dhief et al. [42]	2021	Adoption of online sequential extreme learning machine for voice pathology detection and classification.
Wasmann et al. [43]	2021	New approaches in computational audiology to advance hearing health care.
Heutink et al. [46]	2020	Multi-scale deep learning framework for cochlea localization, segmentation, and analysis on ultra-high-resolution CT images.
Pham et al. [22]	2021	Development of EAR-UNet, a deep learning-based approach for segmentation of tympanic membranes from otoscopic images.
Amanian et al. [24]	2023	Review of AI evolution and applications in rhinology.
Liu et al. [25]	2022	Use of 3D convolutional neural networks and MRI for classifying inverted papilloma malignant transformation.
Fortune-Ely et al. [48]	2023	Future prospects of AI in facial plastic surgery.
Song D et al. [19]	2023	Systematic review of AI technology for diagnosing middle ear diseases using image-based methods.
Parsel et al. [31]	2021	Differentiation of clinical patterns in rhinologic disease.
Beswick et al. [33]	2020	Narrative review on the utility of image guidance in endoscopic sinus surgery.
Campbell et al. [37]	2021	Exploration of the potential of anterior robotic skull base surgery.
Bieck et al. [35]	2020	Language-based translation and prediction of surgical navigation steps for endoscopic wayfinding assistance.
Ogawa et al. [27]	2021	CT texture analysis utility in differentiating olfactory neuroblastoma from sinonasal squamous cell carcinoma.
Ma et al. [15]	2023	Method for preprocessing auditory brainstem response data for automatic classification of hearing loss patients.
Khan et al. [16]	2020	Automatic detection of tympanic membrane and middle ear infections from oto-endoscopic images using convolutional neural networks.
Swaminathan et al. [47]	2023	3D VR rehabilitation therapy for vertigo due to peripheral vestibular dysfunction.

Limitations

This narrative review has some limitations. Firstly, the search strategy was not exhaustive, leading to the subjective bias inherent in the selection and interpretation of literature. There were certain articles and recent studies that could not be included due to restrictions to access. It was possible to describe only selective topics in this study as otorhinolaryngology is a vast and evolving field. The conclusions drawn are based on a selective set of studies, which may affect the generalizability of the findings. Additionally, while the review aims to provide a comprehensive overview, it may not cover all aspects of the topic in depth due to the narrative approach.

## Conclusions

This review amalgamates the various branches of AI and their distinct applications within otology, rhinology, and laryngology. It elucidates the progression of AI, its present-day implementations, and the prospective advancements that lie ahead. Given that otorhinolaryngology is a dynamically evolving field, the integration of AI is likewise in its nascent stages and future endeavors hold the scope of very promising developments, which will not only widen the horizons of otorhinolaryngologists but also have the potential to make people's lives easier and better.

## References

[1] Bur AM, Shew M, New J (2019). Artificial intelligence for the otolaryngologist: a state of the art review. Otolaryngol Head Neck Surg.

[2] (2024). New Gen Apps: Artificial intelligence vs machine learning vs data science. https://www.newgenapps.com/blog/artificial-intelligence-vs-machine-learning-vs-data-science.

[3] van Gerven M, Bohte S (2017). Artificial neural networks as models of neural information processing. Front Comput Neurosci.

[4] You E, Lin V, Mijovic T, Eskander A, Crowson MG (2020). Artificial intelligence applications in otology: a state of the art review. Otolaryngol Head Neck Surg.

[5] Mueller HG, Hornsby BW, Weber JE (2008). Using trainable hearing aids to examine real-world preferred gain. J Am Acad Audiol.

[6] Aldaz G, Puria S, Leifer LJ (2016). Smartphone-based system for learning and inferring hearing aid settings. J Am Acad Audiol.

[7] Wolfgang K (2019). Artificial intelligence and machine learning: pushing new boundaries in hearing technology. Hear Res.

[8] Mira E, Buizza A, Magenes G, Manfrin M, Schmid R (1990). Expert systems as a diagnostic aid in otoneurology. ORL J Otorhinolaryngol Relat Spec.

[9] Kentala E, Pyykkö I, Auramo Y, Laurikkala J, Juhola M (1999). Otoneurological expert system for vertigo. Acta Otolaryngol.

[10] Laurikkala JP, Kentala EL, Juhola M, Pyvkkö IV (2001). A novel machine learning program applied to discover otological diagnoses. Scand Audiol Suppl.

[11] Exarchos TP, Rigas G, Bibas A (2016). Mining balance disorders' data for the development of diagnostic decision support systems. Comput Biol Med.

[12] Yeh SC, Huang MC, Wang PC, Fang TY, Su MC, Tsai PY, Rizzo A (2014). Machine learning-based assessment tool for imbalance and vestibular dysfunction with virtual reality rehabilitation system. Comput Methods Programs Biomed.

[13] Sandhya D, Holi M, Soundararajan K (2016). Classification of brainstem auditory evoked potentials using artificial neural network based on time and frequency domain features. Clin Eng.

[14] McKearney RM, MacKinnon RC (2019). Objective auditory brainstem response classification using machine learning. Int J Audiol.

[15] Ma J, Seo JH, Moon IJ (2023). Auditory brainstem response data preprocessing method for the automatic classification of hearing loss patients. Diagnostics (Basel).

[16] Khan MA, Kwon S, Choo J (2020). Automatic detection of tympanic membrane and middle ear infection from oto-endoscopic images via convolutional neural networks. Neural Netw.

[17] Myburgh HC, Jose S, Swanepoel DW, Laurent C (2018). Towards low cost automated smartphone- and cloud-based otitis media diagnosis. Biomed Signal Process Control.

[18] Norouzi A, Rahim MSM, Altameem A, Saba T, Rad AE, Rehman A, Uddin M (2014). Medical image segmentation methods, algorithms, and applications. IETE Tech Rev.

[19] Song D, Kim T, Lee Y, Kim J (2023). Image-based artificial intelligence technology for diagnosing middle ear diseases: a systematic review. J Clin Med.

[20] Hsu CY, Chen YS, Hwang JH, Liu TC (2004). A computer program to calculate the size of tympanic membrane perforations. Clin Otolaryngol Allied Sci.

[21] Ibekwe TS, Adeosun AA, Nwaorgu OG (2009). Quantitative analysis of tympanic membrane perforation: a simple and reliable method. J Laryngol Otol.

[22] Pham VT, Tran TT, Wang PC, Chen PY, Lo MT (2021). EAR-UNet: a deep learning-based approach for segmentation of tympanic membranes from otoscopic images. Artif Intell Med.

[23] Shie CK, Chang HT, Fan FC, Chen CJ, Fang TY, Wang PC (2014). A hybrid feature-based segmentation and classification system for the computer aided self-diagnosis of otitis media. Annu Int Conf IEEE Eng Med Biol Soc.

[24] Amanian A, Heffernan A, Ishii M, Creighton FX, Thamboo A (2023). The evolution and application of artificial intelligence in rhinology: a state of the art review. Otolaryngol Head Neck Surg.

[25] Liu GS, Yang A, Kim D (2022). Deep learning classification of inverted papilloma malignant transformation using 3D convolutional neural networks and magnetic resonance imaging. Int Forum Allergy Rhinol.

[26] Li C, Jing B, Ke L (2018). Development and validation of an endoscopic images-based deep learning model for detection with nasopharyngeal malignancies. Cancer Commun (Lond).

[27] Ogawa M, Osaga S, Shiraki N (2021). Utility of CT texture analysis to differentiate olfactory neuroblastoma from sinonasal squamous cell carcinoma. Sci Rep.

[28] Ramkumar S, Ranjbar S, Ning S (2017). MRI-based texture analysis to differentiate sinonasal squamous cell carcinoma from inverted papilloma. AJNR Am J Neuroradiol.

[29] Chowdhury NI, Smith TL, Chandra RK, Turner JH (2019). Automated classification of osteomeatal complex inflammation on computed tomography using convolutional neural networks. Int Forum Allergy Rhinol.

[30] LeCun Y, Bengio Y, Hinton G (2015). Deep learning. Nature.

[31] Parsel SM, Riley CA, Todd CA, Thomas AJ, McCoul ED (2021). Differentiation of clinical patterns associated with rhinologic disease. Am J Rhinol Allergy.

[32] Divekar R, Patel N, Jin J (2015). Symptom-based clustering in chronic rhinosinusitis relates to history of aspirin sensitivity and postsurgical outcomes. J Allergy Clin Immunol Pract.

[33] Beswick DM, Ramakrishnan VR (2020). The utility of image guidance in endoscopic sinus surgery: a narrative review. JAMA Otolaryngol Head Neck Surg.

[34] Reiter A, Leonard S, Sinha A, Ishii M, Taylor RH, Hager GD (2016). Endoscopic-CT: learning-based photometric reconstruction for endoscopic sinus surgery. Proc SPIE Int Soc Opt Eng.

[35] Bieck R, Heuermann K, Pirlich M, Neumann J, Neumuth T (2020). Language-based translation and prediction of surgical navigation steps for endoscopic wayfinding assistance in minimally invasive surgery. Int J Comput Assist Radiol Surg.

[36] Steinhart H, Bumm K, Wurm J, Vogele M, Iro H (2004). Surgical application of a new robotic system for paranasal sinus surgery. Ann Otol Rhinol Laryngol.

[37] Campbell RG, Harvey RJ (2021). How close are we to anterior robotic skull base surgery?. Curr Opin Otolaryngol Head Neck Surg.

[38] Żurek M, Jasak K, Niemczyk K, Rzepakowska A (2022). Artificial intelligence in laryngeal endoscopy: systematic review and meta-analysis. J Clin Med.

[39] Witt DR, Chen H, Mielens JD, McAvoy KE, Zhang F, Hoffman MR, Jiang JJ (2014). Detection of chronic laryngitis due to laryngopharyngeal reflux using color and texture analysis of laryngoscopic images. J Voice.

[40] Cho WK, Choi SH (2022). Comparison of convolutional neural network models for determination of vocal fold normality in laryngoscopic images. J Voice.

[41] Ur-Rehman M, Shafique A, Azhar QU, Jamal SS, Gheraibia Y, Usman AB (2024). Voice disorder detection using machine learning algorithms: an application in speech and language pathology. Eng Appl Artif Intell.

[42] Al-Dhief F, Mat Baki M, Abdul Latiff NM, Nik Abd Malik NN, Sabri N, Albadr M, Mahyuddin N (2021). Voice pathology detection and classification by adopting online sequential extreme learning machine. IEEE Access.

[43] Wasmann JA, Lanting CP, Huinck WJ (2021). Computational audiology: new approaches to advance hearing health care in the digital age. Ear Hear.

[44] Verhulst S, Altoè A, Vasilkov V (2018). Computational modeling of the human auditory periphery: auditory-nerve responses, evoked potentials and hearing loss. Hear Res.

[45] Hildebrand MS, DeLuca AP, Taylor KR (2009). A contemporary review of AudioGene audioprofiling: a machine-based candidate gene prediction tool for autosomal dominant nonsyndromic hearing loss. Laryngoscope.

[46] Heutink F, Koch V, Verbist B (2020). Multi-Scale deep learning framework for cochlea localization, segmentation and analysis on clinical ultra-high-resolution CT images. Comput Methods Programs Biomed.

[47] Swaminathan B, Shanmugam VU, Shanmugam R, Prabhash PR, Siddiqi M, Divya PS (2023). 3D virtual reality rehabilitation therapy for patients with vertigo due to peripheral vestibular dysfunction. Indian J Otolaryngol Head Neck Surg.

[48] Fortune-Ely M, Achanta M, Song MS (2024). The future of artificial intelligence in facial plastic surgery. JPRAS Open.

[49] Verde L, De Pietro G, Alrashoud M, Ghoneim A, Al-mutib K, Sannino G (2019). Leveraging artificial intelligence to improve voice disorder identification through the use of a reliable mobile app. IEEE Access.

